# Analysis of Associated Factors and Construction of Risk Prediction Models for Frailty in Hospitalized Older Adults Living With HIV: Protocol for a Prospective Observational Study

**DOI:** 10.2196/84271

**Published:** 2026-02-19

**Authors:** Fan Li, Chaoying Xie, Fang Xiang

**Affiliations:** 1 School of Nursing, University of South China, Hengyang, China; 2 The First Hospital of Changsha, Changsha First Hospital, Kaifu District, Changsha, 410000, China, 86 13975131394; 3 Nursing Department, The Affiliated Changsha Hospital of Xiangya School of Medicine, Central South University, China

**Keywords:** people living with HIV, frailty, risk factors, predictive models, theory of unpleasant symptoms

## Abstract

**Background:**

The aging trend of people living with HIV or AIDS in China is increasing day by day. Frailty is a common condition among older adults living with HIV or AIDS and represents a significant cause of poor prognosis, including falls, decreased quality of life, increased mortality, and potentially prolonged hospital stays. Consequently, early frailty screening in this population holds important clinical significance.

**Objective:**

This study aims to describe the theoretical basis, research objectives, and implementation plan of a prospective observational study. It will focus on investigating the current status of frailty syndrome in hospitalized older adults living with HIV or AIDS, while simultaneously exploring the development of a clinically applicable risk prediction model.

**Methods:**

This study is an ongoing single-center prospective observational study, with a plan to recruit at least 556 hospitalized older adults living with HIV or AIDS (n=445 for development and n=111 for validation). According to the theory of unpleasant symptoms, candidate predictors are categorized into physiological factors (including sociodemographic factors, disease-related influencing factors, sleep, nutrition, and neurocognitive function), psychological factors (including anxiety and depression status), and environmental factors (including social support status). Potential predictors are screened using univariate analysis and least absolute shrinkage and selection operator regression to identify variables for final model inclusion. Model construction and validation employ 3 standard machine learning algorithms: logistic regression, random forest, and support vector machine. Model performance will be evaluated by reporting accuracy, precision, sensitivity, specificity, and the area under the curve.

**Results:**

This study is conducted at a designated infectious disease hospital in Changsha, Hunan Province, China. Participant recruitment commenced on December 22, 2024, and as of December 5, 2025, a total of 603 patients have been enrolled. The primary study findings are anticipated to be published in August 2026.

**Conclusions:**

The findings of this study are expected to provide clinicians in the department of infectious diseases with a convenient tool for frailty risk prediction, thereby enabling early intervention and ultimately improving the long-term health status and quality of life of people living with HIV.

## Introduction

With the promotion and application of highly active antiretroviral therapy, the treatment prospect of AIDS has been significantly improved. Highly active antiretroviral therapy effectively suppresses viral load, controls disease progression in people living with HIV, prolongs survival, and enhances quality of life. The increased survival rate has led to a growing population of people living with HIV or AIDS aged ≥50 years [1]. Consequently, the Centers for Disease Control and Prevention in the United States defines individuals aged ≥50 years with HIV as the “older adult population” rather than the commonly defined population aged ≥60 years [2]. Older adults living with HIV or AIDS face not only age-related disease risks but also chronic HIV-associated conditions and synergistic interactions between HIV and non-HIV comorbidities [2]. Chronic immune damage and inflammation contribute to declining physical function and resilience, predisposing them to frailty—a dynamic geriatric syndrome. The weakened state can lead to a higher risk of adverse events for people living with HIV or AIDS, which is an independent risk factor for new chronic diseases, falls, cognitive decline, multidrug treatment, hospitalization, loss of independence, and increased mortality [3]. Thus, early identification of frailty and implementation of targeted preventive measures are critically important.

Frailty represents a complex state. Strain’s study analyzed neuroimaging markers (including diffusion tensor imaging and cerebral blood flow), revealing that frailty in people living with HIV or AIDS correlates with structural and functional brain alterations, specifically impaired psychomotor speed and executive function performance [4]. HIV-infected individuals who are in a frailty state exhibit atrophy in the anterior cingulate cortex and cingulate cortex regions, which is associated with a decrease in their quality of life. A multidimensional study of HIV-infected adults at the University of California also confirmed this result, showing that frailty has a significant negative impact on the health-related quality of life of people living with HIV or AIDS, and the impact on people living with HIV or AIDS is significantly stronger than that on uninfected adults [5]. In addition, statistical analysis of clinical events following baseline frailty assessment elevated cardiovascular disease risk in frail older adults living with HIV or AIDS (incidence rate ratio of 3.83), followed by new-onset diabetes and orthopedic disorders [6].

The impact of frailty on cognitive function in older adults living with HIV or AIDS is increasingly recognized. HIV-associated neurocognitive disorders (HAND) are one of the most common complications of HIV-related nervous system diseases. According to the diagnostic criteria of the US HIV Neurobehavioral Research Center in 2007, HAND can be divided into asymptomatic neurocognitive impairment, HIV-related mild neurocognitive disorder, and HIV-related dementia [7]. Research has shown that the incidence of HAND in people living with HIV or AIDS over 50 years old is as high as 40.5% to 54.8% [8,9], and there is a synergistic effect between cognitive impairment and frailty [10], that is, frailty can lead to a decline in cognitive function and a higher risk of cognitive impairment. Conversely, impaired cognitive function can also exacerbate the process of frailty. Through a 1-year follow-up study of 60 people living with HIV or AIDS, half of the participants experienced varying degrees of cognitive impairment after the follow-up, with higher levels of frailty indicating a greater risk of cognitive impairment [11]. Notably, both prefrailty and frailty status significantly correlate with HAND, especially mild neurocognitive disorder, further confirming that frailty is an important cause of HAND occurrence [12].

Currently, unidimensional frailty assessment remains predominant in research involving people living with HIV or AIDS, while research on multidimensional frailty status is still insufficient, and the factors leading to multidimensional frailty in hospitalized older adults living with HIV or AIDS are not yet clear. Therefore, we will explore multidimensional frailty as the main outcome. The theory of unpleasant symptoms (TOUS), as a mid-level theory, is commonly used to establish methodological methods and analyze results, or as a theoretical framework for analyzing influencing factors [13,14]. The theory suggests that factors that affect symptoms can be divided into 3 parts: physiological, psychological, and environmental. The three interact and influence each other, jointly affecting disease symptoms and influencing the outcome of symptoms. Guided by TOUS, we categorized potential frailty factors in hospitalized older adults living with HIV or AIDS into physiological, psychological, and environmental domains based on literature review and clinical practice. Ultimately, all included potential influencing factors were identified and finalized through online expert consultation.

Early-stage frailty in older adults living with HIV or AIDS is often relatively insidious. However, to our knowledge, there is relatively little research and development in China on frailty prediction tools for hospitalized population. To address these gaps, we aim to develop and validate a prediction model using 3 machine learning algorithms. By comparing their performance, we seek to enhance risk prediction accuracy, ultimately improving patient prognosis and health outcomes.

This prospective observational study aims to (1) determine the prevalence of multidimensional frailty among hospitalized people living with HIV or AIDS aged ≥50 years, (2) identify factors associated with frailty guided by the TOUS, and (3) develop and validate risk prediction models.

## Methods

### Ethical Considerations

This prospective observational study protocol has been approved by the Medical Ethics Office of Changsha First Hospital (approval number: Ethics Committee: [2025] Express Review [Clinical Documents] No.12) and the Medical Ethics Committee of University of South China (approval number: 2024HLSC025) and registered on ClinicalTrials.gov (ChiCTR2500103387) to ensure transparency and compliance with best practices.

Written informed consent from all participants is required before participating in the study. During the research, all personal data collected (such as identity information, date of birth, medical payment, health information, etc) will be encoded and replaced to protect the identity of participants and be protected under applicable laws. Only researchers and authorized personnel can access these materials. The research results will be reported and disseminated through open and transparent peer-reviewed journals and conference reports.

This study is a noncommercial academic research study and does not provide any direct financial compensation to the participants. All participants receive professional personalized health guidance and counseling based on their examination results, which is the main benefit of their participation in this study.

### Participants and Qualifications

The inclusion and exclusion criteria are presented in Textbox 1.

Textbox 1. **Inclusion and exclusion criteria for a prospective observational study.**

**Inclusion Criteria**

1. People living with HIV or AIDS, who are diagnosed according to the HIV/AIDS virus infection diagnosis industry standard (WS 293-2019) [15]
2. Age ≥50 years
3. Clear awareness and ability to cooperate in completing investigations and testing
4. Voluntary participation and provision of informed consent

**Exclusion Criteria**

1. Physical functional impairments such as severe language communication barriers, hearing impairments, or visual impairments that hinder normal assessment
2. Hemiplegia
3. Refusal to sign the informed consent form

### Patient Recruitment

All eligible patients will be recruited within 24 to 48 hours after admission. Trained researchers will determine potential participants based on inclusion and exclusion criteria and explain this observational study to patients and their families. After patients confirm and sign informed consent forms, they will be included in this study. The examination results and related data of all participants will be subject to strict confidentiality management to ensure the privacy and security of patients. Participants have the right to withdraw from the study in any form at any stage. We plan to recruit at least 556 hospitalized older adults living with HIV or AIDS (n=445 for development and n=111 for validation). The research evaluation will be completed during the patient’s hospitalization period.

### Sample Size

Frailty occurrence in people living with HIV or AIDS represents a binary outcome. Previous studies reported a frailty incidence of 59.3% in hospitalized older adults living with HIV or AIDS, which was assessed using the FRIDE frailty scale [16]. Due to the current lack of a frailty prediction model for HIV/AIDS patients, it is not possible to determine *R*^2^ through previous models. Based on the proportion of outcome events, the maximum *R*^2^ (*R*^2^ cs_max) is calculated to be 0.7416. Assuming that the new model can explain 20% of the variability, the expected *R*^2^ is 0.1483 (=0.7416×20%). We expect the predictive model to include 5 to 10 predictor variables, based on “pmsampsize(type=‘b’, csrsquared=0.1483, parameters=10 , prevalence=0.593).” In this formula, csrsquared represents the ability of the expected prediction model to explain outcome variability; parameters represent the total number of candidate variables expected to be included; and prevalence refers to the prevalence of frailty events in older people living with HIV or AIDS in China based on previous research. The calculation indicates that a minimum sample size of 556 is required. A total of 330 events were expected, corresponding to 33 events per predictor variable. The above results are calculated using the pmsampsize package in R Statistical Software (version 4.4.0) [17].

### Definition of Outcomes

At present, the frailty assessment methods adopted in various domestic studies are inconsistent, and there are significant differences in research results. Most studies use a single physiological dimension frailty scale (such as frailty phenotype). We believe that frailty phenotype, as a universal frailty assessment tool including core indicators such as weight loss and perceived fatigue, may be affected by HIV-specific factors. For example, a decline in immune system function leads to the occurrence of opportunistic infections, as well as nausea, vomiting, diarrhea, and nutrient absorption disorders caused by the side effects of most antiviral drugs, which makes people living with HIV or AIDS more prone to weight loss. In addition, most antiretroviral drugs cause patients to experience fatigue and exhaustion, which may exacerbate physical inactivity. Current research on multidimensional frailty is expanding. Global studies have confirmed the strong applicability of the Tilburg Frailty Indicator in people living with HIV or AIDS [18,19]. Its comprehensive advantage in assessing frailty across physical, psychological, and social domains may render it more suitable for the objectives of this study than unidimensional tools.

The main outcome of this study is the presence of frailty among people living with HIV or AIDS. We use the Tilburg Frailty Indicator for reporting, developed by Gobbens et al [20] and localized by Li et al [21]. The scale consists of 15 items, with physiological dimensions including 8 items: physical health, natural weight loss, walking difficulties, balance, vision, hearing, grip strength, and fatigue; the psychological dimension includes 4 items: memory, depression, anxiety, and coping ability; and the social dimension includes 3 items: living alone, social relationships, and social support. If the patient’s current condition meets the description of 5 or more items, it indicates that the patient has frailty. The final assessment results of the scale will be confirmed by 2 specialists in infection and immunology.

### Prediction Variables

#### Determination of Candidate Variables

The selection of predictive variables for this prediction model is based on a review of literature and consensus opinions by an expert group, taking into account its practicality in clinical settings. Possible clinically relevant variables will be selected as candidate predictors of frailty and evaluated after the patient’s admission examination is completed. Through 3 group discussions and 1 online expert consultation meeting, a total of 31 candidate predictor variables were identified and classified into physiological, psychological, and environmental factors based on TOUS. The variables to be explored are detailed in the following subsections.

#### Physiological Factors

The sociodemographic data assessed included age, gender, marital status, place of residence, education level, work situation, average monthly income, medical payment method, height, weight, BMI, smoking history, alcohol consumption history, and exercise status [22-25].

Disease-related conditions include time of HIV diagnosis, start time of antiviral treatment, route of infection, and the types of diseases that have been diagnosed at present and other associated complications (heart failure, hypertension, cerebrovascular disease, diabetes, moderate or severe liver disease, moderate or severe kidney disease, chronic obstructive pulmonary disease, and tumors) [26-29].

The Pittsburgh Sleep Quality Index (PSQI) developed by Buysse is used for collecting sleep data [30,31]. It includes 7 dimensions: sleep quality, bedtime, sleep duration, sleep efficiency, sleep disorders, hypnotic drugs, and daytime dysfunction, totaling 18 items. Each dimension is scored from 0 to 3 points, with 0 indicating the absence of sleep disorders, 1 indicating mild disorders, 2 indicating moderate disorders, and 3 indicating the presence of severe disorders. The total score ranges from 0 to 21, with a PSQI >7 indicating sleep problems.

Nutritional status is assessed using the Mini Nutritional Assessment-Short Form scale developed by Rubenstein and consists of 6 items, including changes in appetite, weight, mobility, activity, mental health status, and BMI index [32,33]. The total score is 0 to 14 points, with <8 points indicating malnutrition, 8 to 11 points indicating a risk of malnutrition, and ≥12 points indicating good nutritional status. Research has shown that this scale has good sensitivity and specificity in China [34].

Neurocognitive function is evaluated using the International HIV Dementia Scale, a rapid screening tool developed by Sacktor et al [35,36,37]. It can screen for psychomotor speed, attention, working memory, executive function, and language. Including 3 tests with a total score of 12, patients with a score of ≤10 should be further evaluated for possible dementia. Studies have shown that the International HIV Dementia Scale has better efficiency in detecting the neurocognitive performance of patients using antiretroviral drugs [38].

#### Psychological Factors

The Hospital Anxiety and Depression Scale is used for anxiety and depression [39,40]. Mainly used for screening anxiety and depression status of patients in hospitals. The Hospital Anxiety and Depression Scale is divided into 7 anxiety items and 7 depression items. Each item is scored from 0 to 3 points, with higher scores indicating more severe anxiety and depression. This scale has good reliability and validity in the application of people living with HIV or AIDS in China [41,42].

#### Social Factors

The Chinese version of the Social Support Rating Scale developed by Xiao [43] is used to measure an individual’s social support status, including three dimensions: objective support, subjective support, and personal utilization of social support [44,45]. There are a total of 10 items, with a total score of 12 to 66 points. The higher the score, the higher the level of social support. A general total score of ≥45 indicates a high level of support, 23 to 44 indicates a moderate level of support, and ≤22 indicates a low level of support. Social Support Rating Scale is widely used in people living with HIV or AIDS [46].

Laboratory examination indicators were HIV load, CD^4^ T lymphocyte count, albumin, C-reactive protein (CRP), interleukin-6 (IL-6), and soluble tumor necrosis factor receptors (sTNFR1 and sTNFR2) [47-50]. It is worth noting that although inflammatory indicators such as IL-6 and sTNFR1/2 are potential predictive factors for hospitalized older adults living with HIV or AIDS, these items do not have clinical applicability due to their noninfectious and immunological routine clinical testing content, and relevant test results cannot be obtained.

### Data Acquisition

Data will be acquired through questionnaires and hospital medical record systems. We use face-to-face, on-site distribution, and questionnaire collection to obtain general patient information (demographic and sociological information, lifestyle habits, and disease-related conditions), frailty scores, PSQI, anxiety and depression scores, nutrition scores, and social support scores. Physiological measurements will be taken on-site. For participants with lower educational levels or who are unable to complete the survey independently, researchers will fill in the questionnaire on their behalf based on their answers and verify them one by one. To ensure that the natural heterogeneity of the population is not disturbed by human factors, the questionnaire collection process will strictly follow the pre-established standard operating procedures.

The relevant laboratory tests will be conducted within 24 to 48 hours after admission. The collection of venous blood samples is strictly carried out by specialized nurses in the field of infection and immunology, following standard operating procedures. This step is included in the routine admission examination, and we do not charge any additional examination fees for it. We will inform patients before conducting the study that we need to use some personal biochemical test data and enter it into the dataset after obtaining consent. Obtain laboratory test indicators, including HIV load, CD^4+^ T lymphocyte count, albumin, and CRP, through the test records in the case record system of Changsha First Hospital. However, this study did not collect inflammatory indicators such as IL-6 and sTNFR1/2, which may affect the in-depth analysis of the mechanism of frailty inflammation. It is recommended to prioritize supplementary verification in future research.

Neurocognitive function assessments will be administered face-to-face by 2 researchers within 48 to 72 hours of admission. The examination is conducted in a separate interview room to avoid different patients in the same ward being informed of the test content in advance, which may affect the results of the neurocognitive function examination.

### Statistical Analysis

SPSS software (version 26.0; IBM Corp) and R Statistical Software (version 4.4.0; R Foundation for Statistical Computing) are used for data analysis. Categorical variables are presented as frequencies and percentages. Normally distributed continuous data are expressed as mean (SD), while nonnormally distributed continuous or ordinal variables are summarized as median (IQR). Group comparisons employed *t* test or one-way ANOVA for normally distributed continuous variables, Mann-Whitney *U* test (2 groups) or Kruskal-Wallis *H* (multiple groups) test for non-normal/ordinal data, and chi-square test for categorical variables. For baseline data, a *P* value <.05 is considered statistically significant, indicating a significant difference in baseline characteristics between the group with frailty and the group without frailty. For missing data, considering the instability of missing values, this study used multiple imputation method (imputation tool: “mice” package in R Statistical Software [version 4.4.0]) to fill in the missing parts. Participants will be excluded if there are 3 or more candidate variables missing. After imputation, sensitivity analysis will be used to validate the plausibility of the Missing at Random assumption and the robustness of the imputation results. By comparing the results of multiple imputation datasets with complete case analysis (ie, removing missing data), we further validate the robustness of our main conclusions.

### Model Development and Validation

The sample size of this study is relatively small. This study uses logistic regression (LR) as the benchmark for performance comparison. LR has certain advantages in handling limited sample sizes and can demonstrate feature coefficients and significance levels. Although random forest (RF) has the risk of overfitting in small sample situations, it has certain advantages in handling complex nonlinear relationships. We will minimize the risk by adjusting decision tree parameters and cross-validation. Support vector machine (SVM) is particularly suitable for handling situations with high feature dimensions but relatively small sample sizes, and its regularization properties help control the risk of overfitting. As one of the tree ensemble algorithms, XGBoost also has the risk of overfitting and relatively complex parameter tuning. Neural networks have high requirements for sample size and poor clinical interpretability for small sample studies. We hope to ensure the robustness of the model to a large extent in this small sample study. For future larger-scale research, more complex machine learning methods will be used.

Randomly split the training set and validation set in a 7:3 ratio, and use the following machine learning algorithms for modeling: LR model, RF, and SVM. Univariate analysis combined with least absolute shrinkage and selection operator regression will be used to screen predictive factors. To address nonlinearity, we will analyze the relationships between relevant factors and HIV frailty risk using restricted cubic splines and incorporate them as spline terms in the model. Restricted cubic splines can also indirectly capture potential interactions between features. Interactions and complex nonlinear decision boundaries will be handled by selecting nonlinear kernel functions in SVM. The primary model performance evaluation metric is accuracy, which represents the proportion of correctly predicted samples in classification tasks. This will be considered alongside precision, recall, *F*_1_-score, and area under the receiver operating characteristic curve for comprehensive assessment. Through integrated comparison, the algorithm achieving optimal balance across these metrics will be selected as the final model for predicting frailty status in people with HIV.

**Figure 1.**
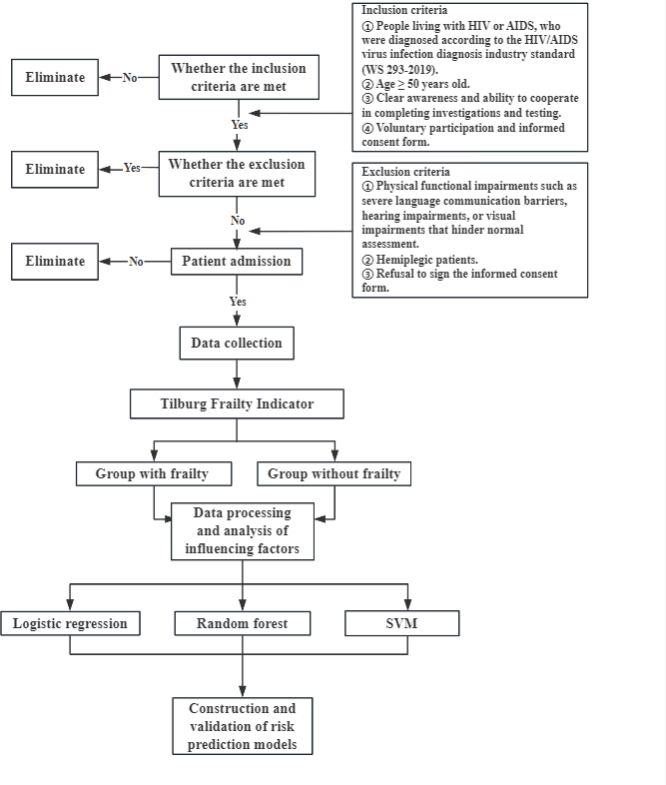
Flow chart [15]. SVM: support vector machine.

To prevent overfitting, the nonlinear modeling effects of all models will be validated via cross-validation. A 3×10-fold cross-validation scheme will be adopted, with each split performed independently, yielding 30 model performance estimates (eg, area under the curve [AUC], *F*_1_-score). Overfitting will be monitored by ensuring that the performance difference between the training set and the cross-validation set remains below a threshold (AUC difference <0.05). During cross-validation, hyperparameters (including max_depth for RF, and C and γ for SVM) will be jointly optimized to select the parameter combination that achieves the best trade-off between fitting efficacy and generalization ability, thereby further reducing overfitting risk. The final model selected through cross-validation will then be evaluated on an independent test set for ultimate performance assessment. For internal validation, we performed bootstrap resampling (n=1000 iterations) to generate training sets matching the original cohort size. Retraining prediction models with these training sets and evaluating performance using the above indicators, we will repeat this process to obtain internal validation performance. External validation will be implemented in prospective observational studies, using external period validation to analyze samples from September 1, 2025 to December 31, 2025. In future research, we plan to collaborate with other institutions to continue external validation. The sample size required for external validation in this study is 1/4 of the modeling sample size, and the minimum sample size for external validation is calculated to be 139 cases (Figure 1).

## Results

This study is conducted at a designated infectious disease hospital, the First Hospital (Infectious Diseases Hospital) of Changsha City, Hunan Province, China, which is one of the professional designated treatment centers for HIV/AIDS in China. It is supported by the Traditional Chinese Medicine Research Program of the Health Commission of Changsha, Hunan Province (no. B202318) and the Hunan Provincial Natural Science Foundation of China (no. 2023JJ60388). The study was planned to be conducted from December 2024 to August 31, 2025, with external validation conducted from September 1, 2025, to December 31, 2025. Recruitment for the study began on December 22, 2024. As of December 5, 2025, a total of 603 patients have been recruited. Currently, no data analysis has been conducted. The primary study results are expected to be announced in August 2026.

## Discussion

Studies indicate a frailty prevalence of up to 12% among people living with HIV or AIDS in China [51], with higher rates observed in hospitalized older adults [16]. With the increasing aging phenomenon of people living with HIV or AIDS, researchers need to further focus on the complex health challenges it brings. The PWH Primary Care Guidelines suggest that it is necessary to conduct frailty screening for individuals aged 50 years and above [52]. This study introduces the principles and design of a registry-based observational study. We hope that the use of this predictive model tool can accurately evaluate whether hospitalized people living with HIV or AIDS have weakened, and we hope that the identification of predictive factors will help health care workers provide preventive care.

Previous studies in China have mostly used frailty assessment tools that focus on physiological dimensions [53,54]. Recent evidence suggests that social vulnerability is particularly prominent in people living with HIV or AIDS [55], and frailty is increasingly reflected in psychological distress and high levels of social isolation [56]. This suggests that using a single physiological dimension evaluation tool may have certain limitations, and the necessity of conducting multidimensional evaluations is evident [18,19]. In addition, current evidence on frailty is mainly derived from cross-sectional studies. Given that frailty is a dynamic evolutionary process (meaning that there are often transitions between different stages of frailty: non-frailty, pre-frailty, and frailty), more cohort studies may be needed in China to validate the risk factors and their dynamic impacts across various dimensions [48].

Considering clinical applicability, some potential relevant biomarker data (IL-6, sTNFR1, sTNFR2) missing from the electronic health data system in this study were not included in the analysis, which may affect the estimation of predictor effects. Studies have shown that inflammatory markers IL-6 and TNFR1 are strongly associated with an increased risk of frailty, with each one-standard-deviation increase in log_10_-IL-6 (OR 1.67, 95% CI 1.20‐2.32) and log_10_ TNFR1 (OR 1.91, 95% CI 1.40‐2.61) [57]. Based on the predictive value of inflammatory markers for frailty in existing research, the absence of such indicators may lead to a decrease in the predictive power (AUC) of the model and result in the estimation of the effects of identified risk factors deviating from the true values. However, the CRP included in this study serves as a systemic inflammatory marker for routine clinical testing, and its expression is induced by IL-6, thus being somewhat correlated with IL-6 levels [58]. This allows CRP to partially compensate for the impact of inflammatory cytokine deficiency on outcomes.

As a regional AIDS treatment center, the First Hospital of Changsha covers many places in Hunan Province and surrounding provinces and cities. Our research is based on this single center design, which may limit its universality and applicability: different regions have certain differences in cultural backgrounds, accessibility of medical care resources, and social support systems [59]. However, it is worth noting that a drug resistance study conducted HIV-1 subtype analysis on the people living with HIV or AIDS of our center [60], showing that the most common subtypes in the region include CRF01_AE and CRF07BC, which is similar to the results reported in multiple other regions of China (Shanxi Province, Guangdong Province, Jiangsu Province, and Shanghai City) [61-64]. This indicates that the virological background of patients in the region may be comparable to a wider population nationwide. In addition, this study is a real-world hospital observational cross-sectional study and cannot establish causal relationships between various related factors and multidimensional frailty. In the future, we plan to conduct multicenter prospective studies to expand the sample size and unify the collection and detection of more potential related biomarkers, including IL-6 and sTNFR1/2. By comparing the performance of the new model with the original model on an independent validation set, we hope to further evaluate the incremental predictive value provided by these biomarkers and verify the causal relationship of related factors.

## Abbreviations

AUC: area under the curve
CRP: C-reactive protein
HAND: HIV-associated neurocognitive disorders
IL-6: interleukin-6
LR: logistic regression
PSQI: Pittsburgh Sleep Quality Index
RF: random forest
SVM: support vector machine
TOUS: theory of unpleasant symptoms
